# The co-expression pattern of VEGFR-2 with indicators related to proliferation, apoptosis, and differentiation of anagen hair follicles

**DOI:** 10.1515/biol-2022-0723

**Published:** 2023-09-20

**Authors:** Ru Dai, Qunye Xu, Zheren Shao, Xianjie Wu

**Affiliations:** Department of Dermatology, Zhejiang University School of Medicine Second Affiliated Hospital, 310009, Hangzhou, China; Department of Dermatology, The First People’s Hospital Daishan, 316261, Zhoushan, China; Department of Plastic Surgery, Zhejiang University School of Medicine Second Affiliated Hospital, 310009, Hangzhou, China

**Keywords:** VEGFR-2, hair follicle, immunofluorescence double staining

## Abstract

An increasing number of studies show that vascular endothelial growth factor is an important regulator of hair growth, and involves in processes of hair follicle development by vascularization. Recently, VEGF receptor-2 (VEGFR-2) has been detected in epithelial cells of hair follicles, indicating that it may have a direct role in the biological activity of hair follicles. To explore how VEGFR-2 regulates hair follicle development, we investigated the co-expression pattern of VEGFR-2 with β-catenin, Bax, Bcl-2, involucrin, AE13 (hair cortex cytokeratin), keratin 16, keratin 14, and Laminin 5 by immunofluorescence double staining in anagen hair follicles of normal human scalp skin. The results of double staining immunofluorescence showed a strong overlapping and similar expression pattern for VEGFR-2 with β-catenin and Bcl-2, and revealing associated expression pattern with involucrin, AE13, keratin 14, keratin 16, and Laminin 5. These results elucidated that VEGFR-2 activation may participate in hair follicle differentiation, proliferation, and apoptosis *in vivo*.

## Graphical abstract

**Figure:**
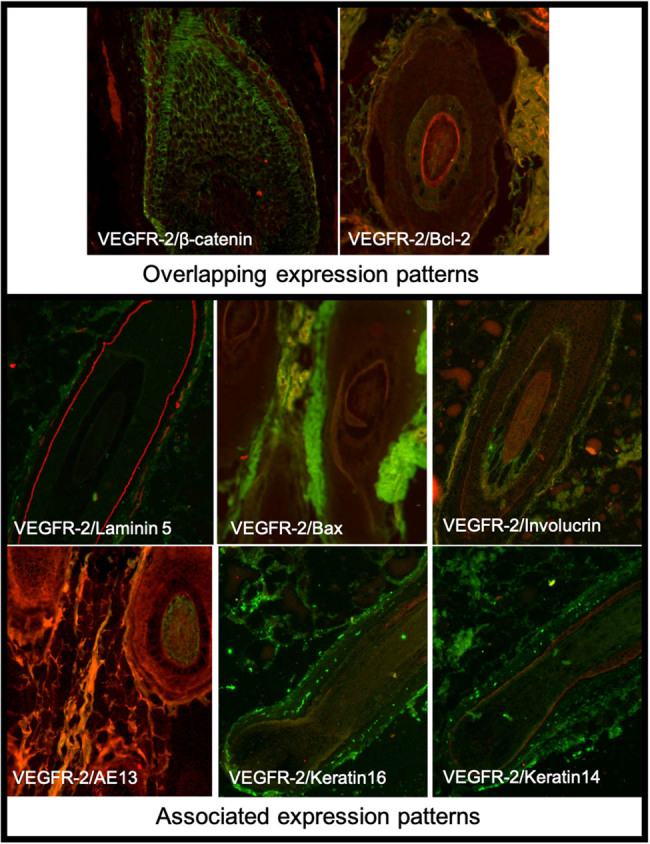

## 1 Introduction

Hair growth is characterized by periodicity, which can be divided into anagen, catagen, and telogen phases. The hair follicle matrix, which is located in the hair bulb surrounding the dermal papilla (DP), is composed of a series of epithelial pluripotent stem cells differentiated from hair follicle stem cells and transitional cells with proliferation and self-renewal abilities [1]. The hair follicle matrix plays an important role in hair follicle growth and hair cycle maintenance. In anagen, hair matrix cells proliferate, migrate, and differentiate to generate the hair shaft. When entering catagen, hair matrix cells stop proliferating while beginning apoptosis, and then, hair follicles enter telogen. During the emergence of the next hair follicle cycle, secondary hair germ cells migrate downward and rewrap around the DP to form the hair matrix. During this cycle, many growth factors and families are involved in the complex regulatory processes [2–4]. Recently, vascular endothelial growth factor (VEGF) was detected in the DP and outer root sheath (ORS) cells during anagen were found to regulate hair growth [5].

During early anagen, the vascularization around hair follicles is significantly enhanced, while the blood vessels around the hair follicles begin to deteriorate in catagen, and then stop; all these phenomena are related to the intensity of VEGF expression in ORS cells [4]. Collagen hydrogel sustained release of VEGF effectively promotes the growth of rat hair follicles, and VEGF is secreted by the DP cells as a signal to ORS cells [6]. The biological effects of VEGF are mediated by its receptors, which consist of VEGF receptor (VEGFR)-1, VEGFR-2, VEGFR-3, neuropilin (NRP)-1, and NRP-2. Among them, the high-affinity VEGFR-2 is considered to be the primary receptor for VEGF and mediates most of its biological activities [7]. Our previous studies have confirmed that VEGFR-2 is expressed not only in hair DP but also in hair follicle epithelium of the ORS, inner root sheath (IRS), hair follicle bulge cells, and dermal sheath cells [8,9]. Moreover, VEGFR-2 was confirmed to vary in the expression on hair follicles during hair cycling [10], and phosphorylated VEGFR-2 was expressed in a whole hair follicle, mainly in the infundibulum basal layer, hair cortex, medulla in the isthmus, and matrix in the hair bulb [11]. VEGF-165 had been shown to promote ORS cell proliferation, migration, and differentiation via VEGFR-2 activation *in vitro* [9]. Therefore, the VEGF/VEGFR-2 pathway may be directly involved in the regulation of hair follicles.

However, whether VEGF and its receptors play an important role in the growth and differentiation of hair follicle keratinocytes *in vivo* is still unclear. In addition, whether the VEGF/VEGFR-2 pathway of hair follicle cells has autocrine or paracrine roles as an important node in the molecular network that regulates hair follicle cycling remains unknown. To clarify these mechanisms, we investigated the co-expression pattern of VEGFR-2 and indicators of proliferation, apoptosis, differentiation, and adhesion in anagen hair follicles of normal adult scalp skin.

## 2 Materials and methods

### 2.1 Specimens

Normal human scalp specimens were obtained from subjects (*n* = 7, mean ± SD age 36.6 ± 11.6 years, range from 18 to 55 years old) with written informed consent at the Department of Plastic Surgery and Dermatology. Scalp specimens were embedded in optimal cutting temperature medium (Miles, Naperville, IL, USA) and stored in liquid nitrogen. This study was approved by the ethics committee of Zhejiang University School of Medicine and conformed to internationally accepted ethical standards.


**Informed consent:** Informed consent has been obtained from all individuals included in this study.
**Ethical approval:** The research related to human use has been complied with all the relevant national regulations, institutional policies and in accordance with the tenets of the Helsinki Declaration, and has been approved by ethics committee of the Second Affiliated Hospital, Zhejiang University School of Medicine

### 2.2 Chemicals and reagents

Anti-Bcl-2, Bax, involucrin, AE13, keratin 14, keratin 16, Laminin5, and β-catenin antibodies were purchased from Santa Cruz Biotechnology (Santa Cruz, CA, USA). Goat anti-mouse-rhodamine and goat anti-rabbit-fluorescein isothiocyanate (FITC) antibodies were purchased from Dako Cytomation (Denmark). 4′,6-Diamidino-2-phenylindole (DAPI) was purchased from Sigma-Aldrich (St Louis, MO, USA). Mouse anti-human VEGFR-2 monoclonal antibody was purchased from Santa Cruz Biotechnology (Santa Cruz, CA, USA), and rabbit anti-human VEGFR-2 polyclonal antibody was purchased from Abcam (Abcam, Cambridge, UK).

### 2.3 *In situ* immunofluorescence double staining

Immunofluorescence co-staining of VEGFR-2 and markers of proliferation, apoptosis, and differentiation was conducted in human anagen hair follicles as described below. Normal scalp specimens embedded in OCT were sectioned at 5–8 µm on a Leica CM 1850 cryostat (Meyer Instruments, Inc., Houston, TX, USA), transferred onto slides, fixed in pre-cooled acetone at −20°C for 20 min, dried at room temperature, and then washed in phosphate buffer solution with Tween-20 (PBST) three times for 5 min each. Membranes were permeabilized using PBST containing 0.1% Triton X-100 at room temperature for 15 min. Slides were washed in PBST three times for 5 min each, and then, 10% bovine serum was added for 1 h at room temperature. The first antibody was added and incubated for 2 h at room temperature. After three washes in PBST for 5 min each, another primary antibody was added, and the samples were incubated for another 2 h at room temperature. Slides were washed in PBST for 5 min three times each, and rhodamine-labeled goat anti-rabbit antibody (1:500) was added and incubated for 2 h in the dark at room temperature. After the samples were washed three times in PBST for 5 min each, FITC-labeled goat anti-rabbit antibody (1:500) was added, and the samples were incubated for a further 2 h in the dark at room temperature. The samples were washed in PBST three times for 5 min each, and 0.5–10 µg/mL DAPI was added for 5 min in the dark. Finally, the slides were washed three times in PBST for 5 min each and then covered. All sections were observed and photographed under a fluorescence microscope. Two types of anti-VEGFR-2 antibody and antibodies to Bcl-2, Bax, involucrin, AE13, keratin 14, keratin 16, Laminin 5, and β-catenin were diluted 1:200. All samples were stained at least 3–4 times to ensure consistent results. The same specimens labeled by rhodamine or FITC were set as negative controls. In our study, the negative controls were hair follicles treated with FITC- and rhodamine-labeled goat anti-rabbit antibodies without adding primary first antibodies for VEGFR-2 and other markers were set as negative controls.

## 3 Results

### 3.1 Immunofluorescence double staining for VEGFR-2 with β-catenin and Laminin 5 in anagen hair follicles

**Figure 1:**
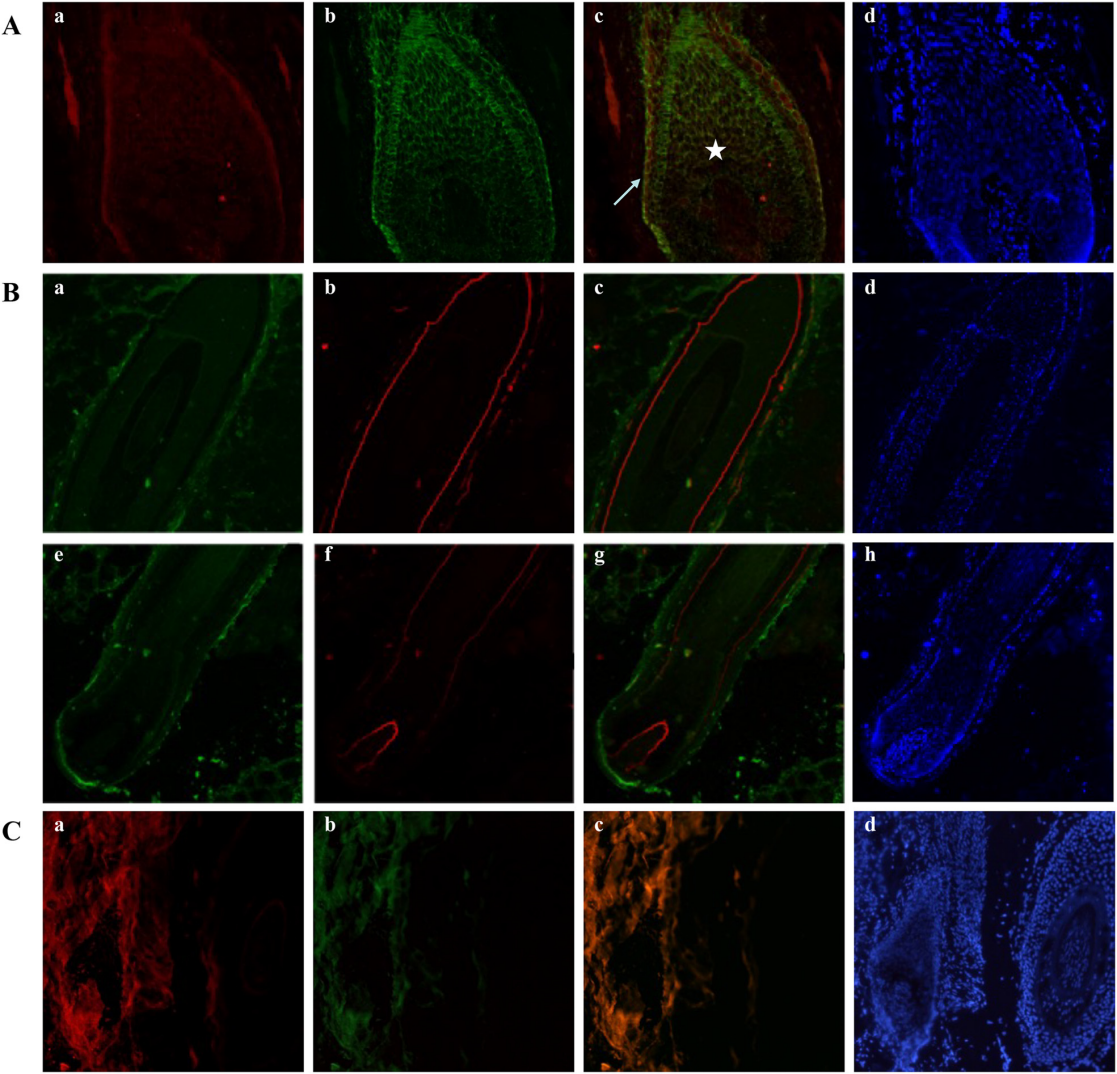
Immunofluorescence double staining for VEGFR-2 with β-catenin and Laminin5 in an anagen hair follicle from human scalp. (A) VEGFR-2 labeled by rhodamine is shown in red (a), and β-catenin labeled by FITC is shown in green (b), and staining for the merge of VEGFR-2 with β-catenin is shown in yellow (c). DAPI dye is shown in blue (d). Staining for VEGFR-2 and β-catenin was increased in the outermost basal cells of the hair bulb (arrow), and co-staining was observed in the hair matrix (star), showing a good overlap. (B) VEGFR-2 labeled by FITC is shown in green (a and e), Laminin 5 labeled by rhodamine is shown in red (b and f), and staining for the merge of VEGFR-2 with Laminin 5 is shown in yellow (c and g). DAPI dye is shown in blue (d and h). Staining for Laminin 5 was mainly observed near the VEGFR-2 positive-staining area in the basement membrane band around the ORS (c) and DP (g). The two proteins were close to each other and showed similar staining, but they did not coincide with each other. (C) Negative control labeled by rhodamine (a), FITC (b), merge of negative control (c), and DAPI (d).

Co-staining for VEGFR-2 and β-catenin was detected in the outermost basal cells of the hair bulb and hair matrix. These proteins were increased in hair bulb basal cells, decreased in the hair matrix and showed strong co-staining and similarity (Figure 1A).

As for VEGFR-2 and Laminin 5, these two proteins exhibited different staining patterns. Laminin 5 staining was observed in the basement membrane band around the ORS and DP, and was mainly adjacent to the VEGFR-2-positive areas. The positively stained areas of these two proteins were close to each other and showed synchronously decreased, but they did not overlap with each other (Figure 1B). Negative controls are presented in Figure 1C.

### 3.2 Immunofluorescence double staining for VEGFR-2 with Bcl-2 and Bax in anagen hair follicles

**Figure 2:**
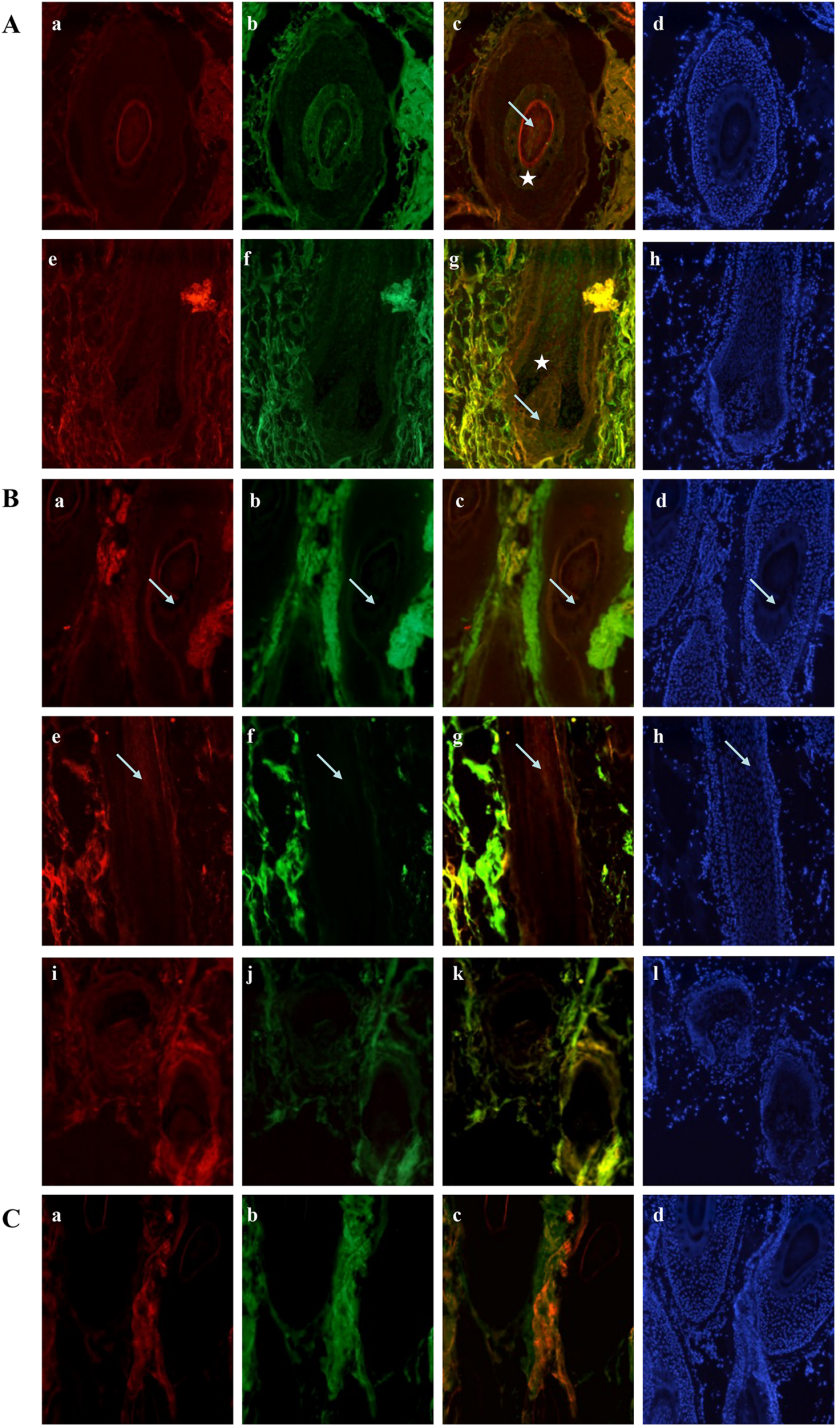
Immunofluorescence double staining for VEGFR-2 with Bcl-2 and Bax in an anagen hair follicle from human scalp. (A) VEGFR-2 labeled by rhodamine is shown in red (a and e), Bcl-2 labeled by FITC is shown in green (b and f), and staining for the merge of VEGFR-2 with Bcl-2 is shown in yellow (c and g). DAPI dye is shown in blue (d and h). Staining for VEGFR-2 and Bcl-2 increased together in the outer basal cells in the hair bulb and the DP (arrow), and decreased similarly in the hair matrix (star). These proteins increased similarly in the medulla and IRS, showing good co-staining. (B) VEGFR-2 labeled by rhodamine is shown in red (a, e, and i), Bax labeled by FITC is shown in green (b, f, and j), and staining for the merge of VEGFR-2 with Bax is shown in yellow (c, g, and k). DAPI dye is shown in blue (d, h, and l). The co-staining pattern for VEGFR-2 and Bax was present only in the IRS in an anagen hair follicle (arrow). (C) Negative control labeled by rhodamine (a), FITC (b), merge of negative control (c), and DAPI (d).

Co-staining for VEGFR-2 and Bcl-2 showed a synchronous pattern with strong co-staining. Both proteins were increased in the outer basal cells in the hair bulb and hair follicle while decreased in the hair matrix. In the isthmus, they also increased uniformly in the medulla and IRS, whereas both deceased in the outer portion of the ORS, showing a good pattern of co-staining (Figure 2A).

Bax was expressed only in the IRS of anagen hair follicles and was not present in the DP and ORS. Therefore, the co-staining of VEGFR-2 and Bax was found only in the IRS (Figure 2B). Negative controls are presented in Figure 2C.

### 3.3 Immunofluorescence double staining for VEGFR-2 with involucrin in anagen hair follicles

**Figure 3:**
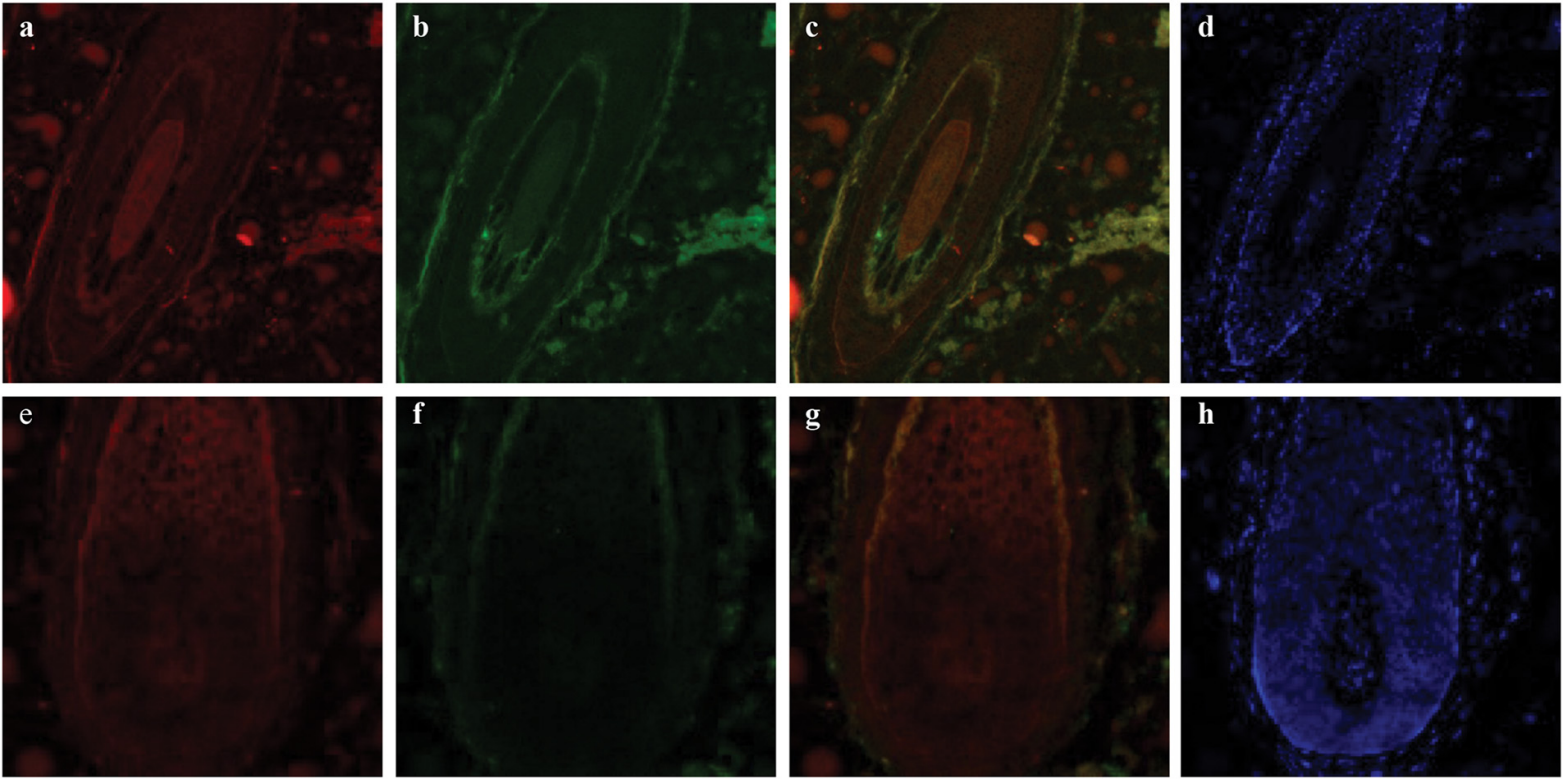
Immunofluorescence double staining for VEGFR-2 with involucrin in an anagen hair follicle from human scalp. VEGFR-2 labeled by rhodamine is shown in red (a and e), involucrin labeled by FITC is shown in green (b and f), and staining for the merge of VEGFR-2 with involucrin is shown in yellow (c and g). DAPI dye is shown in blue (d and h). Staining for VEGFR-2 and involucrin increased jointly in the cortex, medulla, and IRS, showing good co-staining. In the hair bulb, staining for VEGFR-2 and involucrin increased similarly in the outermost basal cells in the hair bulb but showed an opposite staining pattern in the hair medulla, as VEGFR-2 increased and involucrin decreased. Negative control is shown in Figures 1C and 2C.

Co-staining analysis showed that VEGFR-2 and involucrin staining increased jointly in the cortex, medulla, and IRS, whereas it decreased in the ORS and showed good similarity and co-staining expression. In the hair bulb, staining for VEGFR-2 and involucrin increased uniformly in the outermost basal cells while showing the opposite expression pattern in the hair medulla, as VEGFR-2 increased while involucrin decreased (Figure 3).

### 3.4 Immunofluorescence double staining for VEGFR-2 with AE13 (hair cortex cytokeratin) in anagen hair follicles

**Figure 4:**
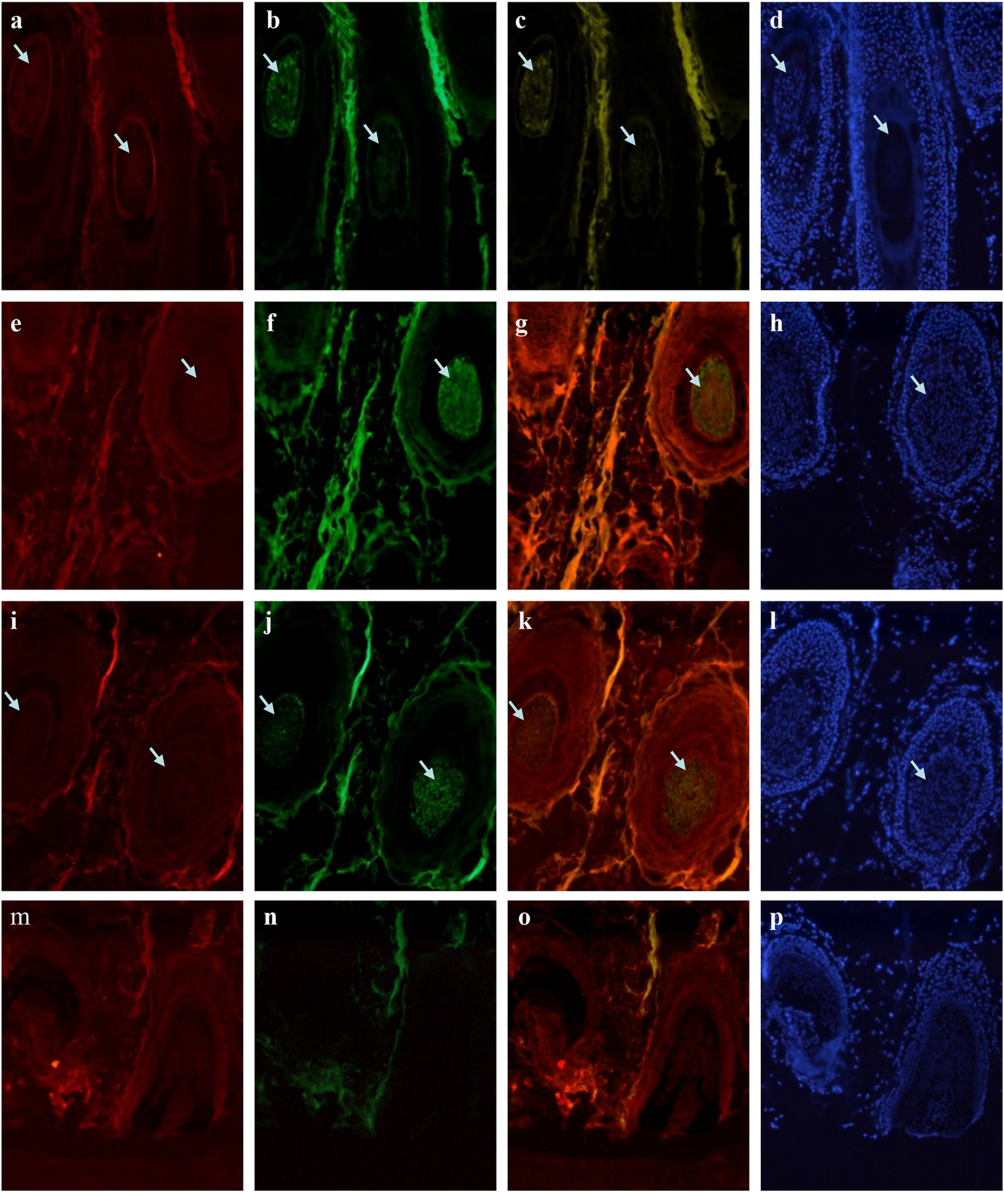
Immunofluorescence double staining for VEGFR-2 with AE13 in an anagen hair follicle from human scalp. VEGFR-2 labeled by rhodamine is shown in red (a, e, i, and m), AE13 labeled by FITC is shown in green (b, f, j, and n), and staining for the merge of VEGFR-2 with AE13 is shown in yellow (c, g, k, and o). DAPI dye is shown in blue (d, h, l, and p). Staining for VEGFR-2 and AE13 increased in the cortex and medulla, showing good co-staining (arrow). Negative control is shown in Figures 1C and 2C.

The staining for VEGFR-2 and AE13 was predominantly restricted in the cortex and medulla, expressing a strong co-staining pattern. In the ORS, the expression of both VEGFR-2 and AE13 was reduced (Figure 4).

### 3.5 Immunofluorescence double staining for VEGFR-2 with keratin 16 in anagen hair follicles

**Figure 5:**
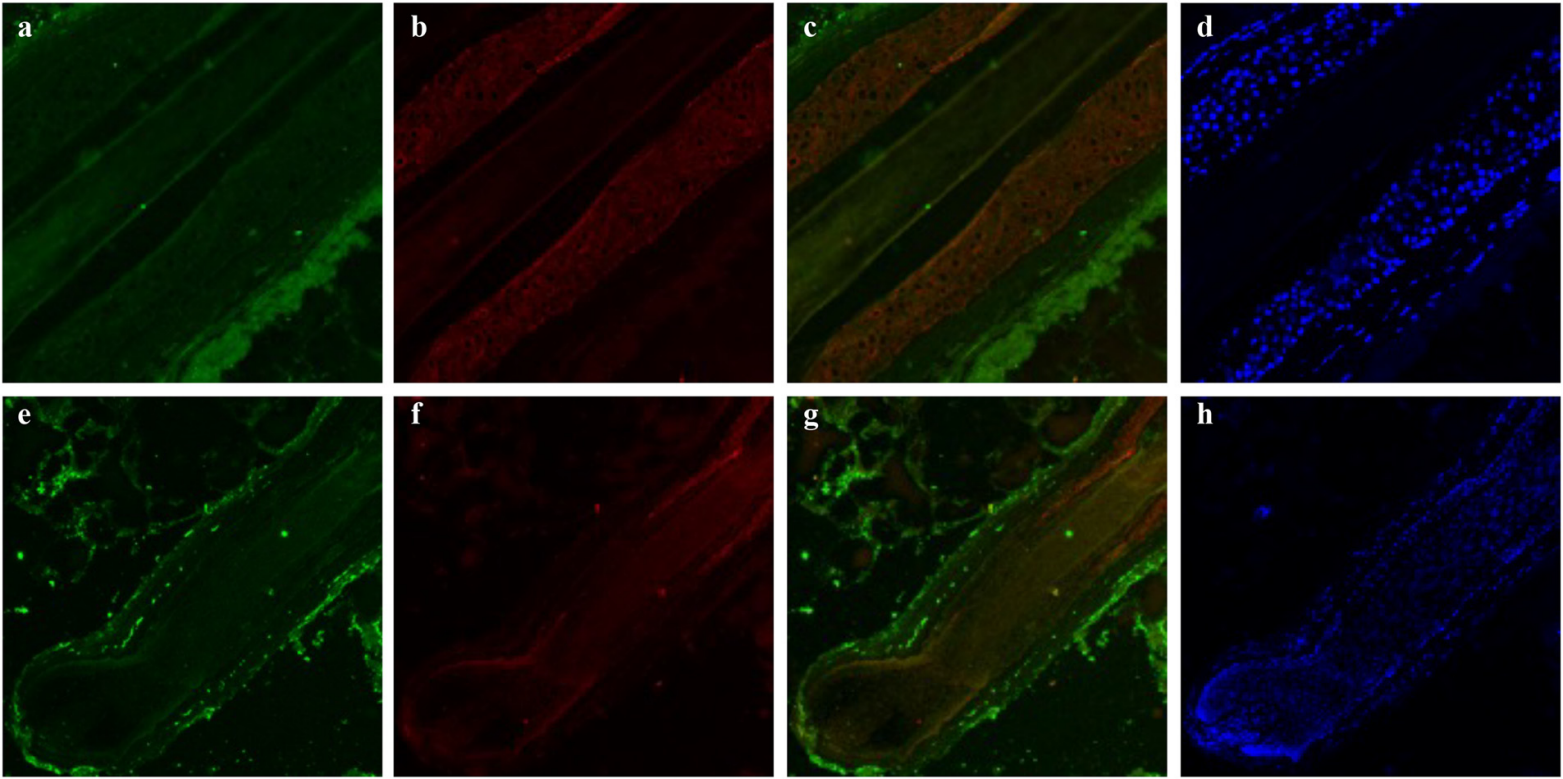
Immunofluorescence double staining for VEGFR-2 with keratin 16 in an anagen hair follicle from human scalp. VEGFR-2 labeled by FITC is shown in green (a and e), keratin 16 labeled by rhodamine is shown in red (b and f), and staining for the merge of VEGFR-2 with keratin 16 is shown in yellow (c and g). DAPI dye is shown in blue (d and h). Staining for VEGFR-2 and keratin 16 increased similarly in the ORS and decreased together in the hair matrix, medulla, cortex, and IRS. Negative control is shown in Figures 1C and 2C.

VEGFR-2 and keratin 16 expression patterns showed close overlap. In the ORS, these two proteins were strongly expressed, while their expression gradually reduced closer to the hair matrix, medulla, cortex, and IRS (Figure 5).

### 3.6 Immunofluorescence double staining for VEGFR-2 with keratin 14 in anagen hair follicles

**Figure 6:**
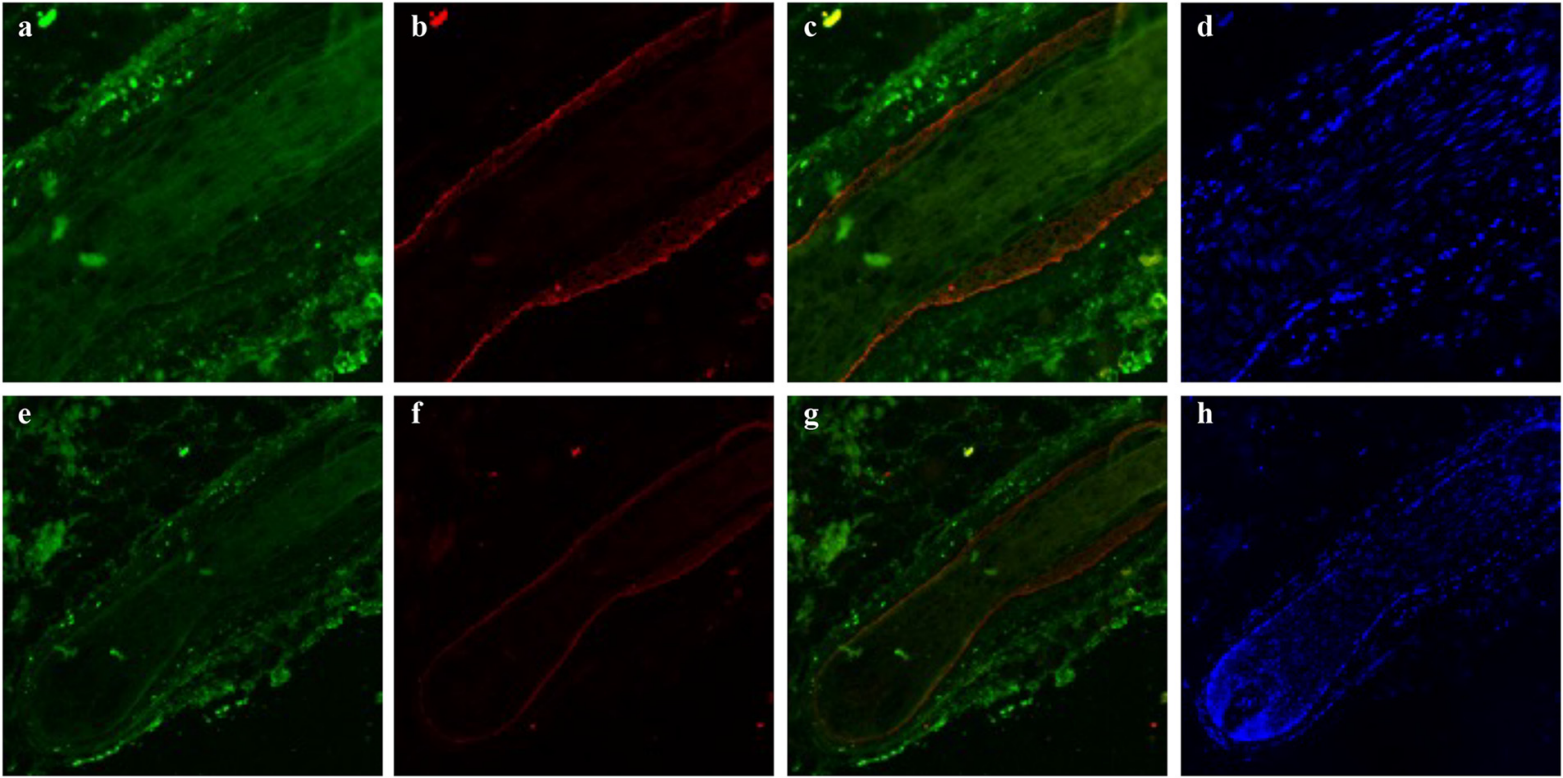
Immunofluorescence double staining for VEGFR-2 with keratin 14 in an anagen hair follicle from human scalp. VEGFR-2 labeled by FITC is shown in green (a and e), keratin 14 labeled by rhodamine is shown in red (b and f), and staining for the merge of VEGFR-2 with keratin 14 is shown in yellow (c and g). DAPI dye is shown in blue (d and h). Staining for VEGFR-2 and keratin14 was mainly observed in the ORS and showed a strong overlap without similar staining style in the hair cortex. Negative control is shown in Figures 1C and 2C.

A co-staining pattern of VEGFR-2 and keratin 14 was observed in the ORS, with strong expression. However, no synchronization was observed in the hair cortex, medulla, IRS, and hair matrix (Figure 6).

## 4 Discussion

The family of VEGF mediates endothelial cell proliferation, vascular permeability, and microvascular formation through specific VEGF receptors [12]. The VEGF family was found to be expressed in some tumor cells, hematopoietic stem cells, neurons, vascular smooth muscle cells, retinal epithelial cells, and retinal ganglion cells [13–16]. In addition, VEGF is the most studied growth factor in the vascularization of the hair follicle, which is produced by DP, keratinocytes of the ORS, and endothelial cells. However, in the bald scalp of patients with hair follicle diseases, the expression of VEGF diminished or even disappears [17]. VEGF could protect human follicle stem cells from 5α-dihydrotestosterone-induced apoptosis down through the PI3K/Akt pathway [18]. Moreover, the VEGF may play a role in prepared platelet-rich plasma and treatment outcomes of hair loss [19].

It is known that the angiogenic effect of VEGF differs according to the VEGFR. VEGFR-1 is pro-proliferative, while VEGFR-2 mediates cell migration [7,20]. VEGF inhibited the adhesion and promoted the proliferation and migration of human epidermal keratinocytes by activating VEGFR-2 [21]. A previous study showed that vitamins and their derivatives could promote hair shaft elongation by activating placental growth factor/VEGFR-1 signaling in follicular matrix cells. Conversely, VEGFR-1 inhibitors markedly reduced the rate of hair shaft elongation that increased by vitamins and their derivatives [22]. Brézillon et al. found that VEGF/VEGFR-2 and HGF/c-Met complexes regulated hair follicle angiogenesis in association with glypican-1 [23]. Glypican-1 might promote the formation of pseudotube and the migration of microvascular endothelial cells. To date, the exact mechanism along with other factors in regulating hair follicles remains unknown. We therefore performed this study to investigate the co-expression patterns of VEGFR-2 with differentiation, proliferation, and apoptosis-related indicators aiming to preliminarily determine the biological significance and function of VEGFR-2 expression in hair follicle epithelium.

Here, we found that VEGFR-2 and Bcl-2 showed similar high expression levels in the basal cells, DP, medulla, and cortex of the hair bulb. However, both proteins are expressed weakly in the hair matrix. Therefore, we suspected that VEGFR-2 activation may be involved in up-regulating of Bcl-2. Both Bax and Bcl-2 are related to apoptosis and belong to the same family of genes, but their roles are contrary. Bax can promote apoptosis, while Bcl-2 inhibits apoptosis, and the ratio of the two determines whether the cell survives or undergoes apoptosis. If Bax is dominant, the cell undergoes apoptosis. On the contrary, if Bcl-2 is dominant, the cell survives [24]. However, co-expression of VEGFR-2 and Bax was found only in the IRS, which is consistent with a previous study. Bax was weakly expressed in the IRS of anagen hair follicles [25], and VEGFR-2 may mediate anti-apoptosis regulation of hair follicle epithelial cells and DP cells in the hair bulb to maintain cell proliferation.

Several studies have suggested that Laminin 5 could promote cell proliferation, adhesion, and migration [26]. In our study, Laminin 5 was detected in the basement membrane band of the isthmus and DP, which was adjacent to positive expressed VEGFR-2. These findings suggest that VEGFR-2 may contribute to Laminin 5 expression in the outer basal layer cells and DP, which was consistent with a previous study that VEGFR-2 activation promoted the proliferation, adhesion, and mobility of ORS cells [9].

Generally, Keratin 16 is expressed in hair follicles and is related to the high proliferative activity of cells [27]. Keratin 14 is mainly located in basal cells and has a strong proliferative ability [28]. In our study, we found that both VEGFR-2 and keratin 16 expressed at a high level in the ORS of the isthmus and hair bulb. As for keratin 14, it was detected in the outer layer of the ORS of the isthmus with a similar expression pattern of VEGFR-2. In addition, these two proteins showed a strong coincident expression in the ORS outer layer of the infundibulum and hair bulb. These results suggest that VEGFR-2 may play a role in activating hair follicle epithelial cell proliferation.

Involucrin and AE13 are markers related to terminal differentiation [29,30]. In the isthmus, the co-staining results for VEGFR-2 with involucrin and AE13 were positive in the cortex and medulla. These are the sites at which hair follicles are terminally differentiated. These results indicated that the VEGFR-2 signal was involved in the differentiation of the cortex and medulla and may be related to the formation and differentiation of the hair shaft. However, VEGFR-2 and involucrin expression showed opposite patterns in the hair bulb. In the matrix and medulla of the bulb, a certain level of proliferation and self-renewal of the cells must be maintained; thereby, inhibiting terminal differentiation is necessary for the biological activity of the hair follicle. The VEGFR-2 signal in the hair follicle matrix and medulla may inhibit the terminal differentiation of these cells and maintain their proliferation and self-renewal abilities to ensure the continuous extension of the hair shaft.

VEGFR-2 and β-catenin staining showed good overlap in the outermost basal cells of the hair bulb, whereas the staining results were opposite in the DP. In our previous study, we found that VEGFR-2 activation was involved in improving β-catenin expression, decreasing homotypic adhesion, and increasing heterotypic adhesion [9]. The indicator of β-catenin plays an important role in hair follicle stem cell activation [31,32]. β-catenin can induce hair follicle formation from hair follicle stem cells to start the hair cycle, and decreased β-catenin expression or a gene blockade mutation can prevent the hair follicle stem cell from developing into a hair follicle and promote development into a sebaceous gland [33]. The accumulation of β-catenin in cells enables the protein to enter the nucleus through the nuclear pores to activate related target genes as transcriptional activators that further regulate the physiological status of stem cells. While β-catenin and E-cadherin are involved in cell connections, they also help maintain stem cell self-stability and can activate stem cell proliferation and differentiation as signal molecules [34]. β-catenin is a central link in the Wnt signaling pathway, and other regulatory roles of many signaling molecules ultimately involve β-catenin. In endothelial and osteo-chondroprogenitor cells, β-catenin acts as a downstream molecule to mediate the physiological function of VEGFR-2 [35,36]. Here, we hypothesized that β-catenin is related to the VEGFR-2 regulation of the hair follicle, but the underlying mechanism requires further investigation.

In conclusion, using an immunofluorescence double staining method, we showed the co-expression patterns of VRGFR2 and various markers of proliferation, apoptosis, and differentiation in the normal adult scalp. In the current work, we imply the clues that activation of VEGFR-2 signaling may be involved in the regulation of hair follicles growth and development. However, our study was a preliminary study evaluating the spatial distribution of VEGFR-2 and compared it with other indicators involved in hair follicle development. In the future, further research should be carefully investigated to explore this work in depth.
